# Immunological reconstitution and infections after alloHCT - a comparison between post-transplantation cyclophosphamide, ATLG and non-ATLG based GvHD prophylaxis

**DOI:** 10.1038/s41409-024-02474-1

**Published:** 2024-11-19

**Authors:** Thomas Meyer, Kristina Maas-Bauer, Ralph Wäsch, Justus Duyster, Robert Zeiser, Jürgen Finke, Claudia Wehr

**Affiliations:** Department of Medicine I/ Hematology, Oncology and Stem Cell Transplantation, Medical Center - University of Freiburg, Faculty of Medicine, University of Freiburg, Freiburg, Germany

**Keywords:** Haematological cancer, Translational research

## Abstract

Immunological reconstitution after allogeneic hematopoietic cell transplantation (alloHCT) is critical for patient survival. We compared short- and long-term immune reconstitution and clinical endpoints in adult recipients of haploidentical or mismatched T cell replete peripheral blood stem cell transplants (PBSCT) with post-transplant cyclophosphamide as GvHD prophylaxis (PTCY, *n* = 68) to: (a) patients receiving matched unrelated grafts and anti-T lymphocyte globulin (ATLG) (MUD/ATLG, *n* = 280); (b) patients with a mismatched donor and ATLG (MM/ATLG, *n* = 54); and (c) recipients of matched related grafts without ATLG (MRD/NoATLG, *n* = 97). PTCY was associated with delayed neutrophil engraftment, low NK-cell counts on day 30 and reduced CD8+ cells on days 60–80. In terms of long-term reconstitution, PTCY recipients demonstrated significantly higher CD4+ counts from day 100–365, primarily derived from naïve T cells. Additionally, B-lymphocyte counts at one year were highest in the PTCY group. Early morbidity and mortality due to infectious complications (viral reactivation, (blood stream) infections) were most frequent in PTCY patients during the first three months. However, beyond three months, no PTCY patient suffered a fatal infection. Our study highlights the pattern of early immunodeficiency followed by robust long-term immune reconstitution in PTCY recipients, identifying critical time periods of risk that could be targeted to optimise patient survival and reduce infectious complications.

## Introduction

Relapse and infections are among the leading causes of death in recipients of allogeneic hematopoietic cell transplantation (alloHCT) [1] of a functional immune system after alloHCT is crucial for the long-term survival of recipients, as it mediates the graft-versus leukaemia effect and controls infections. However, these benefits must be carefully balanced against the risk of graft-versus host disease (GvHD). Both immune reconstitution and GvHD risk are influenced by numerous factors, including donor and recipient age and sex, graft source, graft manipulation, thymic function, prior therapies, GvHD prophylaxis, the occurrence of GvHD and the need for immunosuppressive therapy. While neutrophil and platelet engraftment typically occurs within the first weeks after alloHCT, lymphocyte recovery in adults takes longer. Natural killer (NK) cell reconstitution happens within the first few months, followed by the recovery of CD8+ lymphocytes, B and CD4+ lymphocytes [2, 3].

Since 2008, the use of post-transplantation cyclophosphamide (PTCY) has emerged as an effective GvHD prophylaxis in haploidentical and mismatched donors, and more recently, in matched related and unrelated donors [1, 4–6]. The influence of PTCY on immune recovery has been primarily studied in the early phase after alloHCT, with findings showing that NK, CD4+, CD8+ lymphocyte counts are low during this period [7–11]. Massoud et al. [10] observed a trend towoard faster CD4+ and B-lymphocyte reconstitution in the PTCY group six months after alloHCT. However, the interpretation of these results is often complicated by heterogeneity within the compared groups, such as differences in donor source (bone marrow vs peripheral blood stem cells (PBSC)) or the additional immunosuppression used in the PTCY and non PTCY groups.

In our large, retrospective, single centre analysis (*n* = 499), we examined the impact of donor type and GvHD prophylaxis with PTCY on lymphocyte reconstitution within the first year after alloHCT in adults. We compared PTCY-based regimens with those based on rabbit anti-T lymphocyte globulin (ATLG) and non-ATLGregimens. Our cohort is highly homogeneous concerning the indication for alloHCT, patient age (adults), stem cell source (PBSC), additional GvHD prophylaxis (all patients received cyclosporine and mycophenolate sodium) and the brand and dosing of ATLG (ATLG-Grafalon ® formerly Fresenius ® 30 mg/kg). The analysis also included correlations between immune reconstitution and clinical outcomes relevant to patient morbidity and mortality such as viral reactivation (EBV, CMV), bloodstream infections, inflammation markers and infection-related mortality. In summary, our findings demonstrate that PTCY induces profound immunodeficiency early after alloHCT but facilitates long-term immune reconstitution.

## Subjects, materials, and methods

### Ethics approval

All included patients consented to scientific analysis of their data; there was no study-specific informed consent. The study and consent procedure was approved by the local ethics committee (EK-FR: 21-1590) and conducted in accordance with the declaration of Helsinki.

### Patient cohort

Patient data were retrospectively collected from adults undergoing alloHCT at our centre between 2015 and 2022. We defined the following groups according to donor type and GvHD prophylaxis: (1) haplo-identical (haplo) or mismatch donor (MM) + PTCY 50 mg/kg/d on day +3 and +4; (2) matched related donor (MRD); (3) matched unrelated donor (MUD) + ATLG Grafalon ® 30 mg/kg (4) MM + ATLG Grafalon ® 30 mg/kg. All patients additionally received cyclosporine and mycophenolate sodium for GvHD prevention. Patients with inborn errors of immunity as underlying disease were excluded. Haplo was defined as a 5–7/10 or 4/8 HLA-mismatch, MM as 8–9/10 HLA-mismatch. GvHD was assessed according to standard criteria [12, 13]. Details on patient and transplant characteristics as well as outcome were extracted from our database. If there were multiple causes of death, relapse was always considered the primary cause of death. If relapse was not present in multiple causes of death including infection, infection was considered the primary cause of death. Concerning patient and disease features we defined anything but a partial remission (PR) or complete remission (CR) recorded before alloHCT as active disease. The following regimens were classified as reduced intensity (RIC): TT/FLU/Treo RIC (TT 5 mg/kg, FLU 45 mg/m², TREOS 30 g/m²), TT/BU/FLU RIC (TT 5 mg/kg, BU 6.4 mg/kg, FLU 60 mg/m²). The following regimes were considered reduced toxicity myeloablative (RTC): FLU/TT/MEL (FLU 120 mg/m², TT 10 mg/kg, MEL 110 mg/m²), TT/BU/FLU IIC (TT 10 mg/kg, BU 6.4 mg/kg, FLU 90 mg/m²), TT/FLU/Treo (TT 10 mg/kg, FLU 90 mg/m², TREOS 30 g/m²), FLU/TBI (FLU 120 mg/m², TBI 82 GY), FLU/BCNU/TT (FLU 90 mg/m², BCNU 300 mg/m², TT 15 mg/kg), and FLU/BCNU/MEL (FLU 120 mg/m², BCNU 300 mg/m², MEL 140 mg/m²), the following regimens myeloablative (MAC): FLU/BU 4 (FLU 120 mg/m², BU 12.8 mg/kg), TT/BU/FLU (MAC, TT 10 mg/kg, BU 9.6 mg/kg, FLU 90 mg/m²), TBI/VP16 (TBI 12 GY, VP16 60 mg/kg), TBI/TT (TBI 12 GY, TT 5 mg/kg), FLU/CY (FLU 90 mg/m², CY 150 mg/kg). To stratify conditioning intensity we used the transplant conditioning intensity (TCI) score [14]. CMV risk stratification was based on serostatus with CMV high risk defined as recipient seropositive, CMV intermediate risk defined as recipient negative and donor positive and CMV low risk as donor and recipient seronegative.

### Inflammation assays, viral reactivation and bloodstream infections

Data on C-reactive protein (CRP) and procalcitonin (PCT) spikes, viral reactivation and bloodstream infections were retrospectively extracted. A CRP spike was defined as a CRP value > 50 mg/dl, a PCT spike as a PCT value > 1 ng/ml. An EBV reactivation was defined as EBV > 3000 IU/ml, a CMV reactivation as CMV > 1000 IU/ml. To be counted as a new unique event the value had to drop below threshold or be non existent before. For the correlation of cell counts with spike events or reactivation, the closest cell count value in time was taken into account. A bloodstream infection was defined as positive blood culture (bacteria or fungi) and counted as a unique event if either a new culture was positive >14 days after the last or if a new strain of pathogen was identified.

### Lymphocyte counts

Lymphocyte subsets were determined via flow cytometry after full blood staining on an FACS CANTO II™ and analysed with FACS Diva™ (Becton Dickinson™, New Jersey, USA, Supplementary Methods). All recorded values one year after alloHCT were analysed using automation. Time points were dynamically grouped: Day 30 results included data from days 23–37, day 60 results days 53–70, day 80 results days 71–90, day 100 days 91–120, day 140 days 121–160, day 180 days 161–210, day 240 days 211–270, day 280 days 271–330, day 360 days 331–419. If multiple recordings for one patient and one time point existed the average cell count was used. To compare overall recovery during the first year post-alloHCT, we calculated area under the curve (AUC) values using the trapezoidal rule based on our line plot data. We then determined the AUC ratio by dividing the three highest values to the smallest value or in case of two values dividing the higher by the smaller value, providing a clear measure of relative recovery over the first year post alloHCT.

### Statistics

Data were analysed using R-Studio™ version 2022.12.0, Microsoft Excel™ version 2401, Inkscape™ version 1.2.2 and python version 3.10. For continuous variables distribution was assessed visually. Group comparisons of data with a Gaussian distribution were performed using a two sided t test, non-normally distributed data was tested using a Wilcoxon rank-sum test. To compare multiple groups, we used the Kruskal–Wallis test. For post hoc analysis to compare individual groups, we utilised Dunn’s test with Holm’s method. For categorical variables, a chi-squared test was used to assess differences. Survival data and event curve were assessed using the “survival” package in R [15, 16]. Survival data and events were assessed using the Kaplan–Meier method and compared using a log-rank test. For survival censoring occurred at loss of follow-up. For event curves data were censored at loss of follow-up, at 2nd alloHCT and at death that was not specified as an event.

The associations between various parameters and outcome were analysed using logistic regression models. The models were determined by performing automated stepwise selection of independent variables using a combination of backward elimination and forward selection. The following independent variables were included: Donor age, donor gender, TCI score, degree of MM, age at alloHCT, gender, disease activity before alloHCT, HCT-CI score, CMV risk, Karnofsky performance index, reaching a threshold of lymphocyte counts before day 100: ≥ 20/µl B lymphocytes, ≥50/µl CD4+, ≥199/µl CD8+, ≥143/µl NK cells, maximum counts of aforementioned lymphocytes before day 100, GvHD prophylaxis groups (MM/ATLG, MRD/NoATLG, MUD/ATLG, PTCY).

## Results

### Patient cohort

Among the 499 patients included, 68 received a haplo (*n* = 37) or MM (*n* = 31) graft with PTCY (PTCY) as GvHD prophylaxis. A total of 97 patients received a graft from an MRD without ATLG (MRD/NoATLG), 280 patients from a MUD with ATLG (MUD/ATLG) and 54 patients from a MM donor with ATLG (MM/ATLG). Baseline characteristics were similar across all groups (Table 1). The median age was 60 years (IQR 50-67 years). Most common disease leading to alloHCT in about half of the patients for each group was acute myeloid leukaemia (AML). High CMV risk was comparable across all four groups (PTCY 59%, MRD/NoATLG 53% and MUD/ATLG 56%, MM/ATLG 63%, *p* > 0.05).

**Table 1:** Pre-transplant patient characteristics.

Characteristics	PtCy		MRD		MUD		MM		*p* value
*n*	68		97		280		54		
Age at allo-HCT (yr), median	59.9		58.9		60.8		60.1		>0.05^a^
Donor age (yr), median	33.0		55.0		27.0		31.5		<0.001^a^
Male, *n* (%)	39	57%	55	57%	156	56%	37	69%	>0.05^b^
Female, *n* (%)	29	43%	42	43%	124	44%	17	31%	>0.05^b^
Donor Gender Male, *n* (%)	46	68%	55	57%	200	71%	35	65%	>0.05^b^
Donor Gender Female, *n* (%)	22	32%	42	43%	80	29%	19	35%	>0.05^b^

Baseline disease, *n* (%)	PtCy		MRD		MUD		MM		
AML, *n* (%)	33	49%	44	45%	140	50%	28	52%	>0.05^b^
MDS, *n* (%)	8	12%	9	9%	22	8%	3	6%	>0.05^b^
MPN, *n* (%)	9	13%	12	12%	36	13%	7	13%	>0.05^b^
ALL, *n* (%)	3	4%	5	5%	21	8%	5	9%	>0.05^b^
Lymphoma, *n* (%)	11	16%	17	18%	34	12%	7	13%	>0.05^b^
MM, *n* (%)	4	6%	10	10%	19	7%	3	6%	>0.05^b^
Other, *n* (%)	0	0%	0	0%	8	3%	1	2%	>0.05^b^
Patient with active disease, *n* (%)	48	71%	64	66%	162	58%	34	63%	>0.05^b^
HCT-CI, mean	2.9		2.8		2.8		2.3		>0.05^a^
Karnofsky Performance Score before Tx, mean	82.6		83.1		85.1		88.0		<0.05^a^

Conditioning regimes	PtCy		MRD		MUD		MM		
Conditioning: RIC	10	15%	20	21%	52	19%	6	11%	>0.05^b^
Conditioning: RTC	53	78%	58	60%	180	64%	34	63%	>0.05^b^
Conditioning: MAC	5	7%	19	20%	48	17%	14	26%	<0.05^b^
Conditioning TCI score, median	2.5		2.5		2.5		2.5		>0.05^a^
TCI risk scheme low, *n* (%)	10	15%	21	22%	64	23%	10	19%	>0.05^b^
TCI risk scheme intermediate, *n* (%)	57	84%	71	73%	207	74%	42	78%	>0.05^b^
TCI risk scheme high, *n* (%)	1	2%	5	5%	9	3%	2	4%	>0.05^b^

CMV, *n* (%)	PtCy		MRD		MUD		MM		
CMV high risk (R+)	40	59%	51	53%	156	56%	34	63%	>0.05^b^
CMV intermediate risk (D+/R−)	9	13%	15	15%	18	6%	7	13%	<0.05^b^
CMV low risk (D-/R-)	19	28%	31	32%	106	38%	13	24%	>0.05^b^

^a^Age at allo-HCT, Donor age, HCT-CI mean, Karnofsky Index before Tx, mean, TCI score, median are continuous variable, a Kruskal–Wallis test was used.

^b^All other variables are categorical; thus, a chi-square test was used to assess significance for event occurrence.

### Prevalence of GvHD and immunosuppression

In order to interpret results on immune reconstitution it is important to consider the prevalence of GvHD and its treatment. Acute GvHD (aGvHD) grade II-IV was most prevalent in MRD/NoATLG with 54% of patients compared to 38% in PTCY group, 29% in MUD/ATLG and 39% in MM/ATLG (*p* < 0.001, Table 2). Moderate or severe chronic GvHD (cGvHD) was observed in 53% in MRD/NoATLG, more than doubling the prevalence of moderate or severe cGvHD in PTCY (24%), MUD/ATLG (16%) and MM/ATLG (17%, *p* < 0.001). This divergence is also reflected in the prevalence of systemic immunosuppressive therapies: 80% of MRD/NoATLG patients received systemic glucocorticosteroids compared to 60% of PTCY, 48% of MUD/ATLG and 57% of MM/ATLG (*p* < 0.001). Second line GvHD treatment with ruxolitinib in the first year after alloHCT was also most common in MRD/NoATLG (MRD/NoATLG 42% vs PTCY 21%, MUD/ATLG 15% and MM/ATLG 22%, *p* < 0.001) (Table 2).

**Table 2:** Post-transplant patient characteristics.

Characteristics	PtCy		MRD		MUD		MM		*p* value
*n*	68		97		280		54		
GvHD, *n* (%)	PtCy		MRD		MUD		MM		
aGvHD > I *n* (%)	26	38%	52	54%	80	29%	21	39%	<0.001^b^
cGvHD moderate or severe (%)	16	24%	51	53%	45	16%	9	17%	<0.001^b^

Immunosuppressive therapies	PtCy		MRD		MUD		MM		
Patient with systemic Steroids, *n* (%)	41	60%	78	80%	133	48%	31	57%	<0.001^b^
Patient with Ruxolitinib, *n* (%)	14	21%	41	42%	41	15%	12	22%	<0.001^b^

Death, *n* (%)	PtCy		MRD		MUD		MM		
Overall deceased, *n* (%)	28	41%	42	43%	88	31%	32	59%	<0.001^b^
Median duration till fatal event (days)	88		197		258		229		<0.05^a^
Death due to Relapse, *n* (%)	9	13%	24	25%	44	16%	13	24%	>0.05^b^
Death due to infection, *n* (%)	11	16%	10	10%	25	9%	12	22%	<0.05^b^
Death due to GvHD, *n* (%)	3	4%	3	3%	7	3%	3	6%	>0.05^b^

EBV and CMV reactivations, *n* (%)	PtCy		MRD		MUD		MM		
CMV reactivation incidence (R+)	17	43%	24	47%	76	49%	28	82%	<0.01b
EBV reactivation incidence	4	6%	3	3%	27	10%	9	17%	<0.05b

^a^For median duration till fatal event (days) as a continuous variable, a Kruskal–Wallis test was used. Post hoc analysis can be found in Fig. 5.

^b^All other variables are categorical; thus, a chi-square test was used to assess significance for event occurrence

### Engraftment, NK- and γδ T-cell reconstitution

**Fig. 1:**
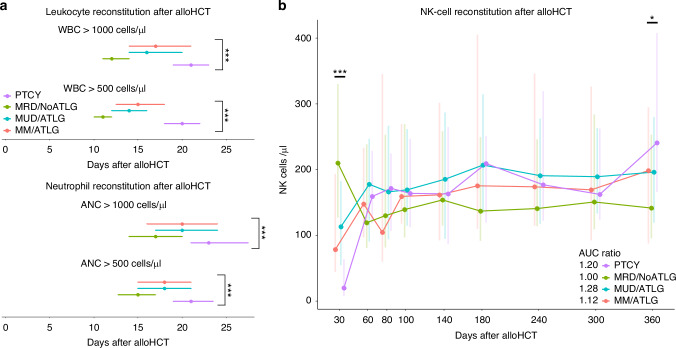
Engraftment and early immune reconstitution. **a** Line graphs display timing of leucocyte engraftment with white blood cell count (WBC) and acute neutrophil count (ANC) for 500 and 1000 cells/µl respectively. **b** Line graph shows natural killer (NK) cell-reconstitution during the first year after alloHCT. Points show median values, lines display interquartile ranges (IQR) 25–75%. * for *p* < 0.05, ** for *p* < 0.01, *** for *p* < 0.001 (Kruskal–Wallis test). Area under the curve (AUC) was calculated for each group and set in relation to the lowest value. AUC are calculated from using the trapezoidal rule on our line plot. For the sake of clarity post hoc analysis are specified in the text.

White blood cell reconstitution was significantly delayed in the PTCY group compared to the three groups without cyclophosphamide after alloHCT (Fig. 1a). In PTCY patients the median time to reach a white blood cell count (WBC) of 1000 cells/µl was 21 days (IQR 19–23 days). WBC recovery was fastest in the MRD/NoATLG group (median 12 days, IQR 11–14 days). The time to leucocyte recovery in the ATLG groups (MUD/ATLG and MM/ATLG) was similar: median time to WBC of 1000 cells/µl was 16 days (IQR 14–20 days) for MUD/ATLG and 17 days (IQR 14–21 days) for MM/ATLG. Post hoc testing showed significant differences (*p* < 0.001) between PTCY and all other groups, as well as between MRD/NoATLG and the ATLG groups (MM/ATLG, MUD/ATLG), but no significant difference between MM/ATLG and MUD/ATLG (*p* > 0.05).

Time to neutrophil engraftment, defined as an absolute neutrophil count (ANC) of >500 cells/µl, was longest in the PTCY and shortest in the MRD/NoATLG group (Fig. 1a). Median time to ANC > 500 cells/µl was 21 days (IQR 19–24 days) for PTCY, 15 days (IQR 13–17 days) for MRD/NoATLG, 18 days (IQR 15–21 days) for MUD/ATLG, and 18 days (IQR 15–21 days) for MM/ATLG. Post hoc testing revealed significant differences (*p* < 0.001) between PTCY and all groups, and between MRD/NoATLG and the ATLG groups (MM/ATLG, MUD/ATLG), but no significant difference between MM/ATLG and MUD/ATLG (*p* > 0.05).

NK cells (CD3-CD16/56+), among the first lymphocytes to recover after alloHCT, also showed delayed recovery in the PTCY group (Fig. 1b). On day 30, median NK cells for PTCY were 20 cells/µl (IQR 8–64), compared to 210 cells/µl (IQR 127–330) in MRD/NoATLG, 113 cells/µl (IQR 55–183) in MUD/ATLG and 79/µl (IQR 45–193) in MM/ATLG (post hoc test: *p* < 0.01 for PTCY vs MM/ATLG, *p* < 0.001 for PTCY vs MUD/ATLG and MRD/NoATLG). By day 60, however, NK-cell counts were comparable across all groups (Fig. 1b). One year post alloHCT, NK-cell counts were highest in the PTCY group at 241 cells/µl (IQR 166–408), compared to 142 cells/µl (IQR 96–254) in MRD/NoATLG, 196 cells/µl (IQR 119–280) in MUD/ATLG, and 198 cells/µl (IQR 88–295) in MM/ATLG (post hoc test: *p* < 0.05 for PTCY vs MUD/ATLG). To compare overall reconstitution in the first year post alloHCT, we analysed the AUC ratio of NK-cell counts until day 365 relative to the minimum value. AUC ratios were comparable across the four groups (AUC ratio: PTCY 1.20, MRD/NoATLG 1.00, MUD/ATLG 1.28, MM/ATLG 1.12). Median time to NK-cell reconstitution, defined as 50% of patients reaching the lower limit of the reference range (>142 cells/µl), was similar between the PTCY and ATLG groups (103 days for PTCY, 89 days for MUD/ATLG, 93 days for MM/ATLG), but delayed compared to MRD/NoATLG (33 days, *p* = 0.051).

**Fig. 2:**
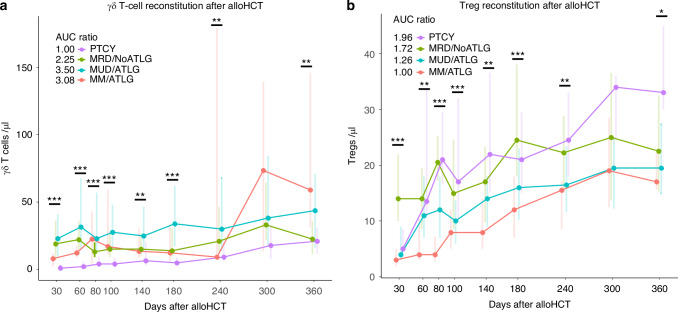
γδ T cells and Treg reconstitution. Line plots (**a** and **b**) show γδ T cell and Treg recovery during first year after alloHCT stratified according to donor type and GvHD prophylaxis. **a** Shows γδ T-cell counts, **b** Treg counts. Points show median, lines display IQR 25–75%. * for *p* < 0.05, ** for *p* < 0.01, *** for *p* < 0.001 (Kruskal–Wallis test). Area under the curve (AUC) was calculated for each group and set in relation to the lowest value. AUC are calculated from using the trapezoidal rule on our line plot. For the sake of clarity post hoc analysis are specified in the text.

γδ T cells mainly reside in non-lymphatic tissue, act in an MHC independent manner and bridge between adaptive and innate immunity. In our cohort, recirculating γδ T cells (TCR γδ+) were persistently low throughout the first year post-alloHCT in the PTCY group, starting with a median of 1 cell/µl (IQR 1–3) on day 30, compared to 19 cells/µl (IQR 7–36) in MRD/NoATLG, 23 cells/µl (IQR 10–41) in MUD/ATLG, and 8 cells/µl (IQR 2–12) in MM/ATLG (post hoc test: *p* < 0.001 for PTCY vs MRD/NoATLG and MUD/ATLG, *p* > 0.05 for PTCY vs MM/ATLG; Fig. 2a). This depletion of γδ T cells in the PTCY group was reflected in the AUC ratios for the first year, with all three groups showing a more than two- to three-fold increase compared to PTCY (AUC ratio: PTCY 1.00, MRD/NoATLG 2.25, MUD/ATLG 3.5, MM/ATLG 3.08).

Given that the PTCY group consisted of both PTCY/MM (*n* = 31) and PTCY/haplo patients (*n* = 37), a subgroup analysis was performed (Supplementary Fig. 1). No significant differences were observed in WBC and ANC recovery times between these subgroups (Supplementary Fig. 1A). However, NK-cell counts on day 30 were significantly lower in the PTCY/haplo (median 17 cells/µl, IQR 7–22) compared to PTCY/MM group (median 53 cells/µl, IQR 10–109, *p* < 0.05, Supplementary Fig. 1B).

### CD8+, CD4+ and B-lymphocyte reconstitution

**Fig. 3:**
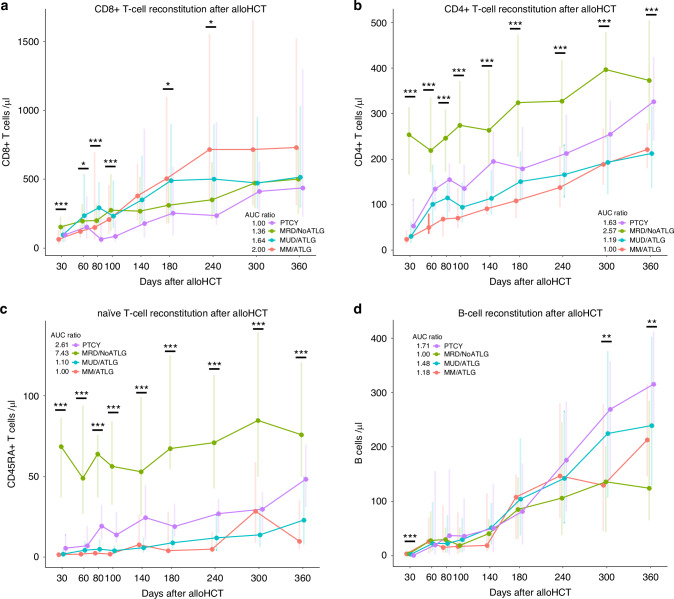
T- and B- lymphocyte reconstitution. Line plots (**a**–**d**) show lymphocyte subpopulation recovery during the first year after alloHCT stratified according to donor type and GvHD prophylaxis. **a** Shows CD8+ T-cell counts, **b** CD4+ T-cell counts, (**c**) CD45RA+/ naïve T-cells counts and (**d**) B-cell counts. Points show median, lines display IQR 25–75%. * for *p* < 0.05, ** for *p* < 0.01, *** for *p* < 0.001 (Kruskal–Wallis test). Area under the curve (AUC) was calculated for each group and set in relation to the lowest value. AUC are calculated from using the trapezoidal rule on our line plot. For the sake of clarity post hoc analysis are specified in the text.

CD8+ (CD3+CD8+) T lymphocytes were comparable between groups during the first two months after alloHCT and after day 100 (Fig. 3a). However, CD8+ cell counts dipped in the PTCY group between day 80–100, compared to the four other groups (d100 count: PTCY median 84 cells/µl, IQR 46–203, MRD/NoATLG 274 cells/µl, IQR 125–536, MUD/ATLG 233 cells/µl, IQR 147-489, MM/ATLG 206 cells/µl, IQR 103-459, post hoc test: *p* < 0.05 PTCY vs MM ATLG, *p* < 0.001 PTCY vs (MRD/ATLG, MUD/ATLG)). CD8+ cell counts in the PTCY group recovered after day 100, becoming comparable to MRD/NoALTG and MUD/NoALTG (post hoc test for all groups *p* > 0.05). This dip and subsequent recovery were also reflected in the AUC ratios with PTCY having the lowest AUC for the first year (PTCY 1.00, MRD/NoATLG 1.36, MUD/ATLG 1.64, MM/ATLG 2.00).

CD4+ lymphocyte counts (CD3+CD4+) were highest in the MRD/NoATLG group during the first year after alloHCT (Fig. 3b). In the PTCY group CD4+ cell counts were consistently the second highest throughout the year, with significantly higher counts from day 100 onward to day 360 post alloHCT compared to the two ATLG groups (Fig. 3b). Day 100 CD4+ median cell count was 136 cells/µl (IQR 91–188) in PTCY compared to 94 cells/µl (IQR 61–149) for MUD/ATLG and 71 cells/µl (IQR 48–137) for MM/ATLG (*p* < 0.05). CD4+ cell counts were highest on day 100 for MRD/NoATLG with 274 cells/µl (IQR 190–372, *p* < 0.001). On day 360 post alloHCT PTCY median CD4+ cell counts were comparable to MRD/NoATLG cell counts (PTCY median 326 cells/µl, IQR 241–425, MRD/NoATLG 373 cells/µl, IQR 263–506, post hoc test: *p* > 0.05). CD4+ cell counts one year after alloHCT were lower in both ATLG groups compared to PTCY (MUD/ATLG median 212 cells/µl, IQR 136–317, MM/ATLG 221cells/µl, IQR 180–279, post hoc test: *p* < 0.05 PTCY vs MM/ATLG, *p* < 0.001 PTCY vs MUD/ATLG). These differences were also evident in the AUC ratios for the first year post alloHCT (PTCY 1.63, MRD/NoATLG 2.57, MUD/ATLG 1.19, MM/ATLG 1.00). The differences in CD4+ counts were further reflected in the median time for 50% of the PTCY population to achieve CD4+ reconstitution, defined as reaching a count of more than 200 CD4+ cells/µl. This milestone was reached on day 211 post alloHCT in the PTCY and on day 33 in the MRD/NoATLG group, compared to more than 300 days in the two ATLG groups (MUD/ATLG 347 days, MM/ATLG 334 days, post hoc test: *p* < 0.01 PTCY vs MUD/ATLG, *p* < 0.001 PTCY vs MM/ATLG). The differences in CD4+ counts were mirrored in CD3+CD4+CD45RA+ lymphocytes, mostly consisting of naïve T-cell counts (Fig. 3c). Naïve T-cell counts in the MRD/NoATLG group exceeded those of the other three groups during the first year after alloHCT. In PTCY patients counts rose from day 80 onwards compared to the two ATLG groups: day 80 PTCY median count for naïve T cells was 20 cells/µl (IQR 11–33) compared to 5 cells/µl (IQR 2–11) for MUD/ATLG and 3 cells/µl (IQR 1–4) for MM/ATLG (post hoc test: *p* < 0.05 PTCY vs MM/ATLG, *p* < 0.01 PTCY vs MUD/ATLG). After one year naïve T-cell counts were constantly rising in PTCY and approaching the level of MRD/NoATLG (PTCY median 49 cells/µl, IQR 31–70, MRD/NoATLG 76 cells/µl, IQR 50–121, post hoc test: *p* > 0.05) and higher compared to MUD/ATLG (median 23 cells/µl, IQR 8–51, post hoc test: *p* = 0.0608). The low naïve T-cell counts within the first year after alloHCT were also evident in the AUC values for this period (PTCY 2.61, MRD/NoATLG 7.43, MUD/ATLG 1.10, MM/ATLG 1.00).

Regulatory T cells (Treg, CD3+CD4+CD25+CD127−) were highest early after transplantation (day 30) in the MRD/NoATLG group, with a median of 14 cells/µl (IQR 10–22, Fig. 2b). In the PTCY group, Treg counts increased from day 30 to day 100, rising from a median of 5 cells/µl (IQR 5–9) to 17 cells/µl (IQR10-32). On day 100, PTCY patients had the highest Treg counts among all four groups (MRD/NoATLG 15 cells/µl, IQR 9–25, MUD/ATLG 10 cells/µl, IQR 6–14, MM/ATLG 8 cells/µl, IQR 5–12, post hoc test: *p* < 0.01 PTCY vs (MM/ATLG, MUD/ATLG), *p* > 0.05 PTCY vs MRD/NoATLG). One year after alloHCT, Treg counts remained higher in the PTCY group (PTCY 33 cells/µl, IQR 30–45, MRD/NoATLG 23 cells/µl, IQR 15–33, MUD/ATLG 20 cells/µl, IQR 15–27, MM/ATLG 17 cells/µl, IQR 16–22, post hoc test: *p* > 0.05). The AUC ratios reflecting Treg reconstitution during the first year were highest for PTCY (1.96) followed by MRD/NoATLG (1.72), MUD/ATLG (1.26) and lowest in MM/ATLG (1.0).

In junction with CD4+T lymphocytes, B lymphocytes are key mediators of adaptive immunity. PTCY facilitated long term B-cell recovery (Fig. 3d). B-cell counts across all four groups were similar for approximately 200 days post alloHCT. One year after alloHCT, PTCY patients had the highest median B-cell count, with 316 cells/µl (IQR 236–413), more than doubling the median count in the MRD/ATLG group with 125 cells/µl (IQR 66–285, post hoc test: *p* < 0.01). Both ATLG groups had lower B-cell counts one year after alloHCT compared to PTCY (MUD/ATLG median 239 cells/µl, IQR 127–403, MM/ATLG 213 cells/µl, IQR 146–315). The higher B-cell counts in the PTCY group were also reflected in the AUC ratios for the first year after alloHCT (PTCY 1.71, MRD/NoATLG 1.00, MUD/ATLG 1.48, MM/ATLG 1.18).

B-cell, CD4+ and naïve CD4+ and CD8+ reconstitution were comparable in the subgroup analysis within the PTCY patients (haplo vs MM) (Supplementary Fig. 1B).

As GvHD and immunosuppressive therapy also affect lymphocyte counts, we stratified lymphocyte subpopulations according to GvHD activity (Supplementary Fig. 2A–D). Patients suffering from aGvHD Grade II or higher and cGvHD moderate or severe had lower B-cell counts starting from day 80 for the entire first year post alloHCT (Supplementary Fig. 2A) as well as lower NK-cell counts from day 30 to day 240 (Supplementary Fig. 2B). This degree of separation concerning the GvHD status could not be observed for CD4+ and CD8+ T cells (Supplementary Fig. 2C, D)

In summary, PTCY patients had high counts of CD4+, naïve CD4+, Tregs and the highest count of B lymphocytes at one year after allo-HCT compared to the other groups.

### Infections

In order to investigate the impact of immune reconstitution on infections we assessed viral reactivation, bloodstream infections and biomarkers of infections in our cohort.

**Fig. 4:**
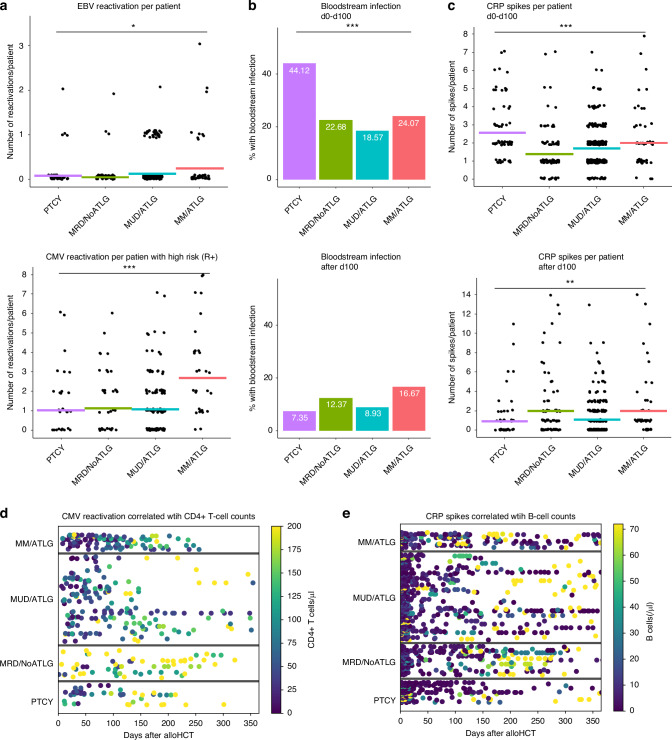
Viral reactivations and bloodstream infections. **a** depicts number of EBV and CMV reactivations per patient with high CMV risk (R+) and group within one year after alloHCT. **b** shows percentage of patients per group who suffered from a bloodstream infection in the first 100 days after alloHCT (upper bar chart) and after 100 days (lower bar chart). **c** depicts CRP spikes per patient and group in the first 100 days (upper point plot) and after day 100 post alloHCT (lower point plot). Points represent single patients with the number of reactivations/ spikes displayed on the y-axis. Coloured lines depict mean per group. **d**, **e** are dotplots for the four groups. **d** depicts CMV reactivation over time as a point with each patient represented as a line, the colour coding represents the CD4+ T-cell counts at reactivation time. **e** depicts CRP spikes with the colour coding representing B-cell counts. * for *p* < 0.05, ** for *p* < 0.01, *** for *p* < 0.001 (Kruskal–Wallis test). For the sake of clarity post hoc analysis are specified in the text.

CMV reactivation rates in CMV high-risk patients (R+) and EBV reactivation rates were comparably low in the PTCY, MRD/NoATLG and MUD/ATLG groups, while MM/ATLG had significantly higher CMV and EBV reactivation rates (Fig. 4a). EBV reactivation prevalence was 17% in the MM/ATLG group, with an average of 0.24 reactivations per patient, compared to 6% in PTCY (0.07 reactivations/patient), 3% in MRD/NoATLG (0.04 reactivations/patient) and 10% in MUD/ATLG (0.12 reactivations/patient) (*p* < 0.05). CMV reactivation in high-risk CMV patients (R+) was highest in MM/ATLG patients (82%) with an average of 2.68 reactivations/patient, while PTCY, MRD/NoATLG and MUD/ATLG had similar CMV reactivation rates (PTCY 43%, MRD/NoATLG, 47%, MUD/ATLG 49%) and average reactivations per patient (PTCY 1.03, MRD/NoATLG 1.14, MUD/ATLG 1.06, post hoc test: *p* < 0.001 MM/ATLG vs (PTCY, MUD/ATLG), *p* < 0.01 MM/ATLG vs MRD/NoATLG). There was no difference between patients with an intermediate (D+/R−) and low CMV risk (D-/R-).

Bloodstream infections and CRP spikes (Fig. 4b, c) were analysed for both early (until day 100 after alloHCT) and late events (after day 100). Early post alloHCT, 44% of PTCY patients had a confirmed bloodstream infections, approximately double the rate of the other three groups: MRD/NoATLG (23%), MUD/ATLG (19%) and MM/ATLG (24%, *p* < 0.05). In contrast, after day 100 the percentage of patients with bloodstream infections was lowest in PTCY and highest in MM/ATLG (PTCY 7%, MRD/NoATLG 12%, MUD/ATLG 9%, MM/ATLG 17%, *p* > 0.05). Pathogens identified included gram-positive bacteria in 104 patients, gram-negative bacteria in 62 patients and fungal pathogens in 17 patients. This difference between early and late infection phases was also reflected in CRP spike differences (Fig. 4c) and PCT spikes across the four groups. The average number of CRP spikes per patient within the first 100 days after alloHCT was significantly higher in PTCY compared to the three other groups and lowest in the MRD/NoATLG (PTCY 2.57, MRD/NoATLG 1.37, MUD/ATLG 1.7, MM/ATLG 2; post hoc test: *p* < 0.05 PTCY vs MM/ATLG, *p* < 0.001 PTCY vs (MUD/ATLG, MRD/NoATLG), *p* < 0.05 MRD vs (MUD/ATLG, MM/ATLG)). Late CRP spikes were more prevalent in MM/ATLG and MRD/NoATLG compared to the other two groups with the lowest average in PTCY (PTCY 0.93, MRD/NoATLG 1.96, MUD/ATLG 1.05, MM/ATLG 1.98 spikes per patient; post hoc test: *p* < 0.05 PTCY vs (MM/ATLG, MRD/NoATLG), *p* < 0.05 MUD/ATLG vs (MM/ATLG, MRD/NoATLG). The average number of PCT spikes per patient had a similar distribution, with highest values for PTCY in the first 100 days post alloHCT and the lowest thereafter (data not shown).

We correlated lymphocyte subpopulations with CMV reactivations (Fig. 4e) and CRP spikes (Fig. 4e). The strongest visual correlation was observed between CD4+ lymphocytes and CMV reactivations and between B cells and CRP spikes. Higher CD4+ cells correlated with fewer CMV infections (Fig. 4d), with a median of 70 CD4+ cells/µl at the time of CMV reactivation within the first year post alloHCT. Figure 4d illustrates the high prevalence of CMV reactivations at low CD4+ lymphocyte counts in the MM/ATLG group. Figure 4e illustrates the correlation between CRP spikes and B-cell reconstitution (Fig. 4e). CRP spikes after day 100 were correlated with low B-cell counts, with a median of only 6 B cells/µlfor the spikes occurring after day 100 to the end of the first year post alloHCT. In PTCY patients, the frequency of CRP spikes decreased after day 100 (Fig. 4e) compared to the other three groups as B-cell counts in the group rose (Fig. 3d).

### Morbidity and mortality

**Fig. 5:**
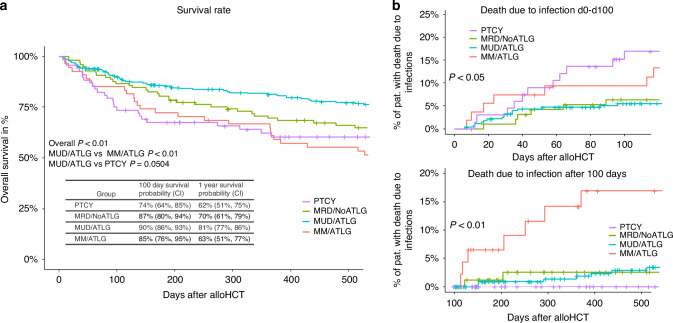
Overall survival and death due to infection. **a** shows the Kaplan–Meier event curve for all four groups. Overall survival in %, ticks mark censoring. Censoring occurred at last follow-up. Table shows 100 day and one year survival probabilities with confidence intervals (CI). **b** depicts event curves for death due to infections before (upper panel) and after (lower panel) day 100 after alloHCT. Censoring is due to loss of follow-up, death not due to infections or 2nd alloHCT. *P* values (log-rank test).

Overall survival (OS) was highest in the MUD/ATLG group (Fig. 5a). Notably, PTCY patients had the lowest OS probability within the first 100 days (PTCY 100-day survival rate: 74% compared to MRD/ATLG 87%, MUD/ATLG 90%, and MM/ATLG 85%). This difference in OS became less pronounced one year after alloHCT (PTCY one-year OS rate 62%, MM/ATLG 63%, MRD/NoATLG 70%, MUD/ATLG 81%, *p* < 0.01; post-hoc analysis cf. Figure 5a). Regarding survival differences based on the degree of mismatch in the PTCY group, survival was worse in the PTCY/haplo subgroup compared to PTCY/MM (Supplementary Fig. 1C).

In general, infections are a leading cause of early mortality (within the first three months) after HCT, while relapse becomes the main cause of mortality later on [1]. Causes of death were comparable between the groups, with infections accounting for approximately 40% of fatalities in both groups. The rate of late death (after day 100) due to progression/relapse of the underlying malignancy was similar in all four groups (PTCY 60%, MRD/NoATLG 62%, MUD/ATLG 62%, MM/ATLG 50%, *p* > 0.05). GvHD free survival (GFS), defined as OS without aGvHD grade II-IV or moderate to severe cGvHD, was also highest in the MUD/ATLG group. However, there was no statistically significant GFS difference between PTCY, MRD/ALTG and MM/ALTG (Supplementary Fig. 2E). We analysed death due to an infectious complication early after alloHCT (day 0–100) and late after alloHCT. Within the first 100 days, the probability of dying due to infectious complications was 17% for PTCY, compared to 9% for MM/ATLG, 6% for MRD/NoATLG and 5% for MUD/ATLG (*p* < 0.05). Thus, the pronounced difference in early survival in PTCY is partly attributable to infectious complications (Fig. 5b). However, after day 100, no PTCY patient who survived thus far died due from an infectious complication. In contrast, MM/ATLG patients who survived beyond 100 days had a 14% probability of dying from infections within one year after alloHCT, while MRD/NoATLG (3% of patients) and MUD/ATLG (2% of patients) had comparable risks for late death due to infections (*p* < 0.01). As we observed a correlation between CRP spikes and B-cell depletion, B-cell depleted patients were also overall more likely to die from late infections. Specifically, 52% of patients who died due to late infections were B-cell depleted, never reaching a threshold of ≥20 cells/µl, compared to only 11% of patients without late infectious fatalities being B-cell depleted (*p* < 0.001).

To determine the factors influencing long-term outcomes, including death and death due to infection after day 100, we performed a multivariate analysis. For predicting death after day 100, the variables determining our stepwise-selected logistic regression models included age at alloHCT, GvHD prophylaxis group MM/ATLG (with belonging to the group as a risk factor), the Karnofsky Performance Score at alloHCT, donor age, TCI score, donor gender, CMV risk intermediate and low, and achieving a threshold of ≥20 B cells/µl in the first 100 days (Supplementary Table 1). For predicting death due to infection after day 100 with multivariate analysis, the factors determining the outcome were age at alloHCT, gender (with male gender as a risk factor), GvHD prophylaxis group MM/ATLG (with belonging to this group as a risk factor), GvHD prophylaxis group PTCY (with PTCY as a protective factor), and the maximum count of B- and CD4+ T-cells reached from day 0–100. B-cell count was a protective factor, while the maximum CD4+ cell count was a risk factor probably due to higher lymphocyte counts in the MRD/NoATLG group. Our multivariate analysis thus confirmed our observation of B-cell reconstitution and not belonging to the MM/ATLG group are associated with better OS and lower risk of death due to infection (Supplementary Table 1). In summary, the early immune deficiency characterised by delayed leucocyte reconstitution and low NK-cell counts in PTCY patients is reflected in a high early rate of infectious complications, leading to an early OS deficit. However, the late immune competence, marked by the reconstitution of CD4+ and B cells after PTCY, correlates with a low rate of late infectious complications with no late fatalities due to infections.

## Discussion

In our single centre cohort of 499 adult alloHCT recipients with haematological malignancies, the one year survival probability was highest in patients receiving a graft from a MUD. The prevalence of acute and chronic GvHD was highest in the MRD/NoATLG group, confirming the protective effect of both ATLG and PTCY against GvHD [5, 17–19]. Besides the lacking protective effect of ATLG or PTCY in the MRD group, the high prevalence of GvHD in recipients of MRD is also influenced by the advanced age of the sibling donors (median age of our cohort 60 years). Consistent with the higher GvHD prevalence, immunosuppressive treatment with steroids or ruxolitinib was most frequent in the MRD/NoATLG group.

In our cohort viral reactivations (EBV, CMV) were far more frequent in the MM/ATLG group, while rates were comparable between PTCY, MUD/ATLG and MRD/NoATLG groups. This difference is influenced by several factors: 1) Letermovir has been approved in 2017 for prophylaxis of CMV reactivation during the first three months after alloHCT. And in more recent years the centre practice has switched from ATLG to PTCY as GvHD prophylaxis in MM grafts [19]. Thus, in our cohort more PTCY patients might have received letermovir prophylaxis than MM/ATLG patients. 2) Lymphocyte reconstitution during the first year after alloHCT was significantly better in PTCY compared to MM/ATLG patients, with more NK cells, naïve CD4+ cells, and B lymphocytes, facilitating better viral control. We could observe a clear correlation of CD4+ depletion and CMV, explaining the vulnerability of the MM/ATLG population. 3) Due to the relatively small sample size of our PTCY cohort (including both MM and haploidentical grafts) it was not possible to assess the effect of the degree of HLA mismatch on the rate of viral reactivations. Little et al. [20] found more CMV and adenovirus infections in recipients of haploidentical grafts compared to MM grafts after PTCY. Rambaldi et al. [8] observed higher CMV reactivation rates in PTCY patients (receiving haplo-identical bone marrow) compared to patients receiving tacrolimus and methotrexate (with HLA-identical PBSC). Due to these confounding factors (different graft source, HLA-matching, GvHD prophylaxis) it is difficult to conclude which factor influenced CMV reactivation. In summary, evidence from the literature suggests that the degree of HLA-mismatch influences the risk of viral reactivation also in the PTCY setting [20] while in our dataset viral reactivation was most prevalent in the MM/ATLG setting both due to confounding factors outlined above and especially the impaired lymphocyte reconstitution compared to PTCY.

Infections and infection-related death within the first 100 days after transplant in our cohort were significantly more frequent in patients receiving PTCY, whereas late fatalities (>100 days after alloHCT) due to infections predominantly occurred in the MM/ATLG group. Overall survival and infection control in our cohort was best in the MUD/ATLG setting. Infectious mortality and the reported frequency of blood stream infections in PTCY of 44% within 100 days and the cumulation of early infectious fatalities after PTCY is in line with reports from other centres [20–24]. One contributing factor to early infection may be the longer time to neutrophil engraftment, a known feature of PTCY [5, 8, 10]. The propensity for infection in PTCY patients was also observed in the randomised, prospective BMT CTN 1703 trial comparing GvHD prophylaxis with PTCY/tacrolimus/methotrexate versus tacrolimus/methotrexate after reduced intensity conditioning: in this setting grade two but not grade three (nor grade four or five) infections were more frequent in the PTCY arm [5]. Importantly, no PTCY patient in our cohort died from infection after day 100, suggesting functional immune reconstitution in these patients. As mentioned above, the highest death rate due to infections was observed in our MM/ATLG group, despite comparable GvHD rates with the PTCY group. Late death from infections was comparably low in PTCY and both MUD/ATLG and MRD/noATLG. We were able to show, that PTCY positively influences lymphocyte reconstitution compared to MM/ATLG, with higher counts of NK, naïve CD4+, CD4+, and B lymphocytes at one year, correlating with a lower late infection burden in the PTCY group. We observed a correlation between CRP levels (as a marker of infection) and B-cell depletion, highlighting the importance of humoral immunity in the PTCY group for late infection control. Naïve CD4+-cell counts were low in MM/ATLG patients one year post alloHCT, accompanied by an inverted CD4+/CD8+ ratio. While NK, total CD4+ and B-lymphocyte counts were comparable to those in the MUD/ATLG group, they were lower than in the PTCY group. This suggests a dysfunctional T-cell compartment and thymic environment in MM/ATLG, not present in MM/haplo PTCY nor MUD/ATLG populations.

In our study, the PTCY group had significantly higher CD4+ counts compared to ATLG-based protocols, consistent with findings by Massoud et al. [10], who reported higher counts half a year after alloHCT. Our one-year follow-up data suggest a durable effect, likely due to a higher count of naïve T cells. Naïve CD4+ cells originate either from the PBSC graft or as recent thymic emigrants from a functional thymic environment. The thymic microenvironment is influenced by the type of conditioning, occurrence of GvHD and use, dose and type of AT(L)G [25–27]. Our data point to a protective role of PTCY in this context. Regulatory T cells have been reported to contribute to the GvHD protective effect of PTCY [28–30], and our data align with the published evidence showing higher Treg counts in the PTCY group three months post-alloHCT compared to ATLG and non-ATLG groups.

In our population NK-cell deficiency in PTCY compared to ATLG and non-ATLG-based groups was observed only early after alloHCT. After day 60, NK-cell numbers were comparable across all groups. This early NK depletion and its swift recovery in PTCY compared to the ATLG and non-ATLG settings was also found by others [8–10]. However, this deficiency in early immunocompetence in our cohort did not lead to more CMV reactivations, as observed by Rambaldi and colleagues [8].

B-lymphocyte counts in our cohort were higher in PTCY patients at one year after alloHCT and similar until approximately day 200 compared to both ATLG and non-ATLG-based protocols. Previously published data [31] have shown tendencies for higher B-cell counts in PTCY compared to ATLG at six months and Khimani et al. showed higher B-lymphocyte counts in PTCY compared to ATLG-free GvHD prophylaxis [32]. Due to the retrospective nature of the study, data on the functional properties of B lymphocytes (e.g immunoglobulin levels) were not available. Early B-lymphocyte development takes place in the bone marrow and later stages maturate in the spleen. Iwamoto et al. have shown that PTCY facilitates early B-lymphocyte development in the bone marrow [33].

Regarding outcome prediction based on lymphocyte reconstitution, both CD4+ [34, 35] and B-cell recovery [36] have been associated with better overall survival. Although our study was primarily descriptive due to the dynamic nature of immune reconstitution, our multivariate analysis for survival showed a correlation between early B-lymphocyte recovery and survival. We did not observe a protective effect of CD4+ cells, which might be due to the fact that our MRD/NoATLG group consistently had high CD4+ cell counts, yet experienced impaired survival compared to the MUD/ATLG group.

In summary, this retrospective study evaluated the immune reconstitution of 499 adult patients receiving PTCY compared to ATLG-based protocols (MUD and MM) and non-ATLG-based protocols (MRD) after T-cell replete PBSCT. We found that early immunological reconstitution was delayed in the PTCY group compared to the other groups leading to a high rate of infectious fatalities. However, late reconstitution in the PTCY group combined the beneficial characteristics of ATLG-based regimens with superior B-lymphocyte reconstitution and the favourable features of non-ATLG-based regimens with robust T-lymphocyte reconstitution. These findings were reflected in the patterns of early infectious complications and late immunocompetence. Future research should focus on the mechanisms of recovery of the thymic and bone marrow environments that influence naïve CD4+ and B-lymphocyte recovery, as well as strategies to prevent early infectious morbidity in PTCY recipients.

## Supplementary information

Supplementary information

## Data Availability

The datasets generated and analysed during the current study are available from the corresponding author on reasonable request.
