# Supplementary material for: Immunological reconstitution and infections after alloHCT - a comparison between post-transplantation cyclophosphamide, ATLG and non-ATLG based GvHD prophylaxis
Source: Bone Marrow Transplant. 2024 Nov 19;60(3):286–96. doi: 10.1038/s41409-024-02474-1 (PMC11893447; doi:10.1038/s41409-024-02474-1)
Supplement: Supplementary file 1 — Supplementary information [file 41409_2024_2474_MOESM1_ESM.docx]

# Supplemental Material


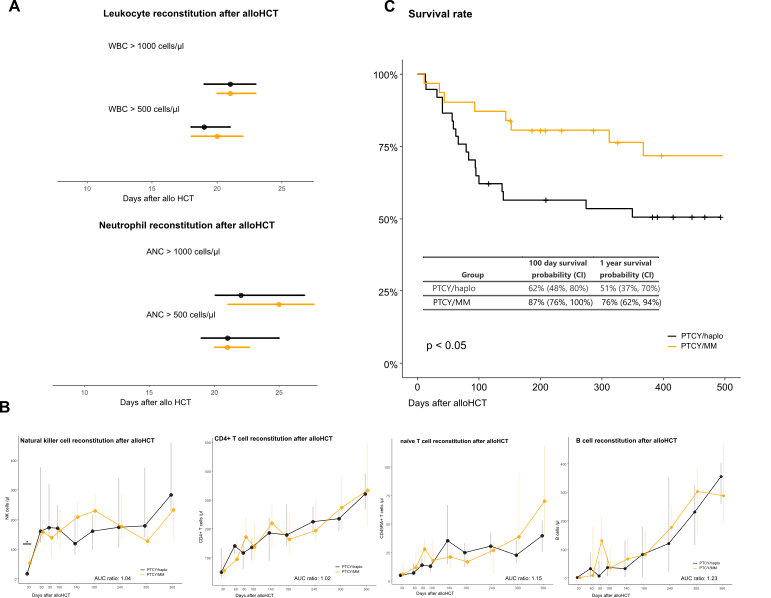


### Supplemental Figure 1: Comparison of survival, engraftment and immunological reconstitution in PTCY haplo and PTCY MM subgroups

(A) Line graphs display timing of leukocyte engraftment with white blood cell count (WBC) and acute neutrophil count (ANC) for PTCY haploidentical (PTCY/haplo) and mismatched PTCY (PTCY/MM) for 500 and 1000 cells/µl respectively. (C) Line plots show lymphocyte subpopulation recovery during the first year after alloHCT for PTCY/haplo vs PTCY/MM. NK-cell counts, CD4+ T-cell counts, CD45RA+/ naïve T-cells counts and B-cell counts are shown. (C) shows the Kaplan-Meier event curve for PTCY/haplo and PTCY/MM. Overall survival in %, ticks mark censoring. Censoring occurred at last follow-up. Table shows 100 day and one year survival probabilities with confidence intervals (CI). Points show median, lines display IQR 25-75%. * for p < 0.05 (Wilcoxon rank-sum test).


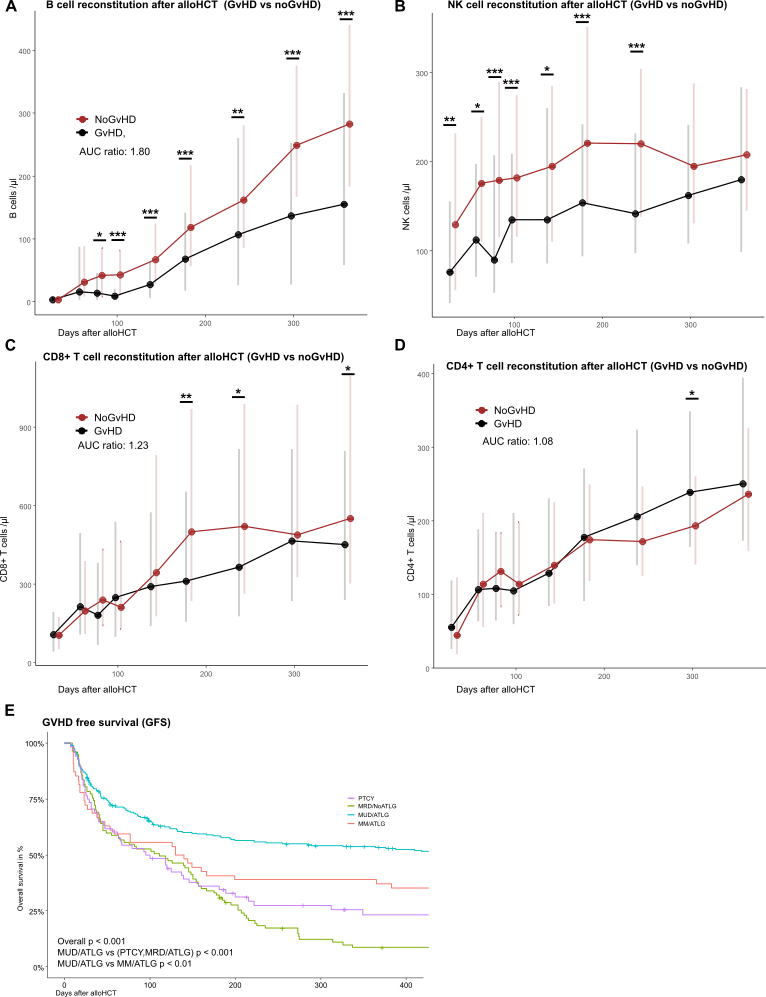


### Supplemental Figure 2: GvHD outcome and reconstitution

1. depicts B-cell reconstitution based on suffering or not suffering from aGvHD > I or cGvHD moderate or severe. Patients change groups from NoGvHD to GvHD with the occurrence of aGvHD > 1 or cGvHD moderate or severe. (B) shows NK-cell recovery based on GvHD status (C) shows CD8+ cell recovery based on GvHD status, (D) shows CD4+ reconstitution based on GvHD status. (E) shows GvHD free survival (GFS) for the four groups, concerning survival with or without aGvHD > I or cGvHD moderate or severe. Points show median, lines display IQR 25-75%. Area under the curve (AUC) ratio is calculated by dividing greater AUC by smaller AUC. AUC are calculated from using the trapezoidal rule on our line plot. * for p < 0.05, ** for p <0.01, *** for p <0.001 (Wilcoxon rank-sum test and log-rank test).

### Supplemental Table 1

| **Prediction of death due to infection after d100 post alloHCT** | | | | | | | |
| --- | --- | --- | --- | --- | --- | --- | --- |
|  | **estimate** | **std.error** | **z-statistic** | **p** | **OR** | **95 % CI lower** | **95 % CI upper** |
| (Intercept) | -2,781 | 1,690 | -1,645 | 0,10 | 0,06 | 0,00 | 1,62 |
| Age at allo-HCT | 0,051 | 0,013 | 3,834 | 0,00 | 1,05 | 1,03 | 1,08 |
| MM/ATLG | 1,509 | 0,355 | 4,252 | 0,00 | 4,52 | 2,27 | 9,18 |
| Karnofsky Performance Score at alloHCT | -0,031 | 0,011 | -2,924 | 0,00 | 0,97 | 0,95 | 0,99 |
| Donor Age | 0,019 | 0,009 | 2,126 | 0,03 | 1,02 | 1,00 | 1,04 |
| TCI score | 0,545 | 0,275 | 1,985 | 0,05 | 1,72 | 1,02 | 3,01 |
| Threshold of ≥20 B cells/µl cells reached (day 0-100) | -0,544 | 0,247 | -2,197 | 0,03 | 0,58 | 0,36 | 0,94 |
| Donor Gender | -0,404 | 0,256 | -1,576 | 0,11 | 0,67 | 0,40 | 1,11 |
| Not CMV risk intermediate/low | -0,435 | 0,247 | -1,763 | 0,08 | 0,65 | 0,40 | 1,05 |
| **Prediction of death due to infection after d100 post alloHCT** | | | | | | | |
|  | **estimate** | **std.error** | **z-statistic** | **p** | **OR** | **95 % CI lower** | **95 % CI upper** |
| (Intercept) | -5,801 | 2,751 | -2,109 | 0,03 | 0,00 | 0,00 | 0,36 |
| Age at allo-HCT | 0,083 | 0,032 | 2,617 | 0,01 | 1,09 | 1,03 | 1,17 |
| MM/ATLG | 2,310 | 0,636 | 3,630 | 0,00 | 10,08 | 2,88 | 36,25 |
| Gender | 1,584 | 0,704 | 2,250 | 0,02 | 4,88 | 1,40 | 23,91 |
| Karnofsky Performance Score at alloHCT | -0,046 | 0,021 | -2,200 | 0,03 | 0,96 | 0,92 | 1,00 |
| Max. number of B cells/µl cells reached (day 0-100) | -0,013 | 0,009 | -1,531 | 0,13 | 0,99 | 0,97 | 1,00 |
| Max. number of CD4+ T cells/µl cells reached (day 0-100) | 0,002 | 0,001 | 1,779 | 0,08 | 1,00 | 1,00 | 1,00 |
| PTCY | -15,729 | 1471,268 | -0,011 | 0,99 | 0,00 | not estimable | 1,75E+33 |

Intercept is the baseline log-odds of the outcome when all predictor variables are zero.

Estimate reflects the change in log-odds per unit change in the predictor. Positive values indicate an increase in the likelihood of the event occurring, in this case, death or death due to infection after day 100.

In our model predicting death due to infection after day 100 post alloHCT, the PTCY value shows perfect separation as no PTCY patient died due to late infections. When we excluded PTCY and the degree of mismatch (which is confounded by PTCY), the other features yielded similar results (data not shown)

### Supplemental Table 2

| **Cell type and day after alloHCT** | **PTCY** | | | **MRD/NoATLG** | | **MUD/ATLG** | | | **MM/ATLG** | | |  |
| --- | --- | --- | --- | --- | --- | --- | --- | --- | --- | --- | --- | --- |
| **NK cells** | **median** | **IQR** | | **median** | **IQR** | **median** | | **IQR** | **median** | | **IQR** | **p value (Kruskal-Wallis test)** |
| day 30 | 20 | (8 - 64) | | 210 | (127 - 330) | 113 | | (55 - 183) | 79 | | (45 - 193) | p < 0.001 |
| day 60 | 160 | (119 - 229) | | 119 | (81 - 239) | 178 | | (91 - 247) | 148 | | (97 - 234) | p > 0.05 |
| day 80 | 172 | (107 - 225) | | 130 | (82 - 254) | 166 | | (94 - 267) | 105 | | (60 - 346) | p > 0.05 |
| day 100 | 164 | (112 - 247) | | 139 | (97 - 269) | 169 | | (115 - 262) | 159 | | (118 - 269) | p > 0.05 |
| day 140 | 163 | (87 - 265) | | 154 | (115 - 258) | 186 | | (95 - 287) | 162 | | (92 - 302) | p > 0.05 |
| day 180 | 209 | (119 - 251) | | 137 | (92 - 249) | 207 | | (134 - 315) | 176 | | (110 - 405) | p > 0.05 |
| day 240 | 177 | (123 - 320) | | 141 | (114 - 246) | 191 | | (117 - 278) | 174 | | (115 - 347) | p > 0.05 |
| day 300 | 163 | (124 - 262) | | 151 | (109 - 284) | 190 | | (133 - 263) | 169 | | (92 - 326) | p > 0.05 |
| day 360 | 241 | (166 - 408) | | 142 | (96 - 254) | 196 | | (119 - 280) | 198 | | (88 - 295) | p < 0.05 |
| **Cell type and date** | **PTCY** | | **MRD/NoATLG** | | | | **MUD/ATLG** | | | **MM/ATLG** | |  |
| **γδ T cells** | **median** | **IQR** | | **median** | **IQR** | **median** | | **IQR** | **median** | | **IQR** | **p value (Kruskal-Wallis test)** |
| day 30 | 1 | (1 - 3) | | 19 | (7 - 36) | 23 | | (10 - 41) | 8 | | (2 - 12) | p < 0.001 |
| day 60 | 2 | (1 - 6) | | 22 | (11 - 36) | 32 | | (15 - 68) | 13 | | (9 - 18) | p < 0.001 |
| day 80 | 4 | (3 - 10) | | 13 | (9 - 20) | 23 | | (14 - 57) | 23 | | (4 - 43) | p < 0.001 |
| day 100 | 4 | (2 - 9) | | 15 | (9 - 29) | 28 | | (16 - 48) | 17 | | (12 - 59) | p < 0.001 |
| day 140 | 7 | (3 - 21) | | 15 | (11 - 27) | 25 | | (14 - 47) | 14 | | (9 - 30) | p < 0.01 |
| day 180 | 5 | (4 - 9) | | 14 | (10 - 31) | 34 | | (17 - 62) | 13 | | (6 - 34) | p < 0.001 |
| day 240 | 9 | (6 - 21) | | 21 | (14 - 47) | 30 | | (15 - 68) | 9 | | (8 - 179) | p < 0.01 |
| day 300 | 18 | (8 - 33) | | 33 | (22 - 65) | 38 | | (18 - 85) | 73 | | (14 - 139) | p > 0.05 |
| day 360 | 21 | (12 - 31) | | 23 | (11 - 36) | 44 | | (16 - 71) | 59 | | (31 - 146) | p < 0.01 |
| **Cell type and date** | **PTCY** | | **MRD/NoATLG** | | | | **MUD/ATLG** | | | **MM/ATLG** | |  |
| **CD8+ T lymphocytes** | **median** | **IQR** | | **median** | **IQR** | **median** | | **IQR** | **median** | | **IQR** | **p value (Kruskal-Wallis test)** |
| day 30 | 97 | (45 - 171) | | 155 | (99 - 226) | 98 | | (44 - 163) | 66 | | (29 - 132) | p < 0.001 |
| day 60 | 154 | (67 - 374) | | 197 | (123 - 321) | 235 | | (127 - 538) | 123 | | (74 - 169) | p < 0.05 |
| day 80 | 66 | (37 - 217) | | 199 | (132 - 318) | 294 | | (169 - 479) | 151 | | (66 - 698) | p < 0.001 |
| day 100 | 84 | (46 - 203) | | 274 | (125 - 536) | 233 | | (147 - 489) | 206 | | (103 - 459) | p < 0.001 |
| day 140 | 179 | (75 - 865) | | 267 | (164 - 520) | 350 | | (193 - 671) | 380 | | (201 - 613) | p > 0.05 |
| day 180 | 253 | (93 - 592) | | 311 | (177 - 524) | 492 | | (230 - 901) | 506 | | (179 - 1101) | p < 0.05 |
| day 240 | 237 | (168 - 918) | | 350 | (212 - 623) | 503 | | (250 - 899) | 717 | | (346 - 1553) | p < 0.05 |
| day 300 | 411 | (246 - 628) | | 472 | (289 - 708) | 474 | | (286 - 953) | 718 | | (309 - 1657) | p > 0.05 |
| day 360 | 438 | (225 - 1301) | | 502 | (249 - 728) | 516 | | (293 - 1032) | 731 | | (312 - 1523) | p > 0.05 |
| **Cell type and date** | **PTCY** | | **MRD/NoATLG** | | | | **MUD/ATLG** | | | **MM/ATLG** | |  |
| **CD4+ T lymphocytes** | **median** | **IQR** | | **median** | **IQR** | **median** | | **IQR** | **median** | | **IQR** | **p value (Kruskal-Wallis test)** |
| day 30 | 53 | (29 - 111) | | 254 | (166 - 314) | 31 | | (15 - 63) | 24 | | (13 - 45) | p < 0.001 |
| day 60 | 135 | (87 - 154) | | 219 | (172 - 335) | 101 | | (51 - 188) | 50 | | (36 - 81) | p < 0.001 |
| day 80 | 155 | (84 - 191) | | 246 | (172 - 309) | 115 | | (67 - 147) | 68 | | (30 - 77) | p < 0.001 |
| day 100 | 136 | (91 - 188) | | 274 | (190 - 372) | 94 | | (61 - 149) | 71 | | (48 - 137) | p < 0.001 |
| day 140 | 195 | (125 - 262) | | 263 | (182 - 395) | 114 | | (78 - 176) | 91 | | (78 - 129) | p < 0.001 |
| day 180 | 179 | (112 - 271) | | 325 | (205 - 473) | 151 | | (107 - 217) | 108 | | (71 - 181) | p < 0.001 |
| day 240 | 213 | (162 - 299) | | 327 | (224 - 418) | 166 | | (117 - 230) | 138 | | (94 - 228) | p < 0.001 |
| day 300 | 255 | (188 - 328) | | 397 | (245 - 479) | 193 | | (123 - 252) | 189 | | (144 - 267) | p < 0.001 |
| day 360 | 326 | (241 - 425) | | 373 | (263 - 506) | 212 | | (136 - 317) | 221 | | (180 - 279) | p < 0.001 |
| **Cell type and date** | **PTCY** | | **MRD/NoATLG** | | | | **MUD/ATLG** | | | **MM/ATLG** | |  |
| **Regulatory T cells** | **median** | **IQR** | | **median** | **IQR** | **median** | | **IQR** | **median** | | **IQR** | **p value (Kruskal-Wallis test)** |
| day 30 | 5 | (5 - 9) | | 14 | (10 - 22) | 4 | | (3 - 9) | 3 | | (2 - 5) | p < 0.001 |
| day 60 | 14 | (11 - 34) | | 14 | (9 - 20) | 11 | | (7 - 18) | 4 | | (3 - 11) | p < 0.01 |
| day 80 | 21 | (17 - 30) | | 21 | (17 - 25) | 12 | | (7 - 18) | 4 | | (3 - 7) | p < 0.001 |
| day 100 | 17 | (10 - 32) | | 15 | (9 - 25) | 10 | | (6 - 14) | 8 | | (5 - 12) | p < 0.001 |
| day 140 | 22 | (15 - 37) | | 17 | (10 - 23) | 14 | | (9 - 18) | 8 | | (5 - 14) | p < 0.01 |
| day 180 | 21 | (15 - 30) | | 25 | (18 - 38) | 16 | | (10 - 23) | 12 | | (7 - 18) | p < 0.001 |
| day 240 | 25 | (18 - 33) | | 22 | (16 - 29) | 17 | | (12 - 23) | 16 | | (9 - 17) | p < 0.01 |
| day 300 | 34 | (25 - 36) | | 25 | (14 - 37) | 20 | | (12 - 25) | 19 | | (13 - 29) | p > 0.05 |
| day 360 | 33 | (30 - 45) | | 23 | (15 - 33) | 20 | | (15 - 27) | 17 | | (16 - 22) | p < 0.05 |
| **Cell type and date** | **PTCY** | | **MRD/NoATLG** | | | | **MUD/ATLG** | | | **MM/ATLG** | |  |
| **B cells** | **median** | **IQR** | | **median** | **IQR** | **median** | | **IQR** | **median** | | **IQR** | **p value (Kruskal-Wallis test)** |
| day 30 | 1 | (1 - 2) | | 4 | (1 - 8) | 3 | | (1 - 7) | 4 | | (2 - 9) | p < 0.001 |
| day 60 | 20 | (2 - 156) | | 28 | (7 - 82) | 23 | | (9 - 99) | 26 | | (4 - 70) | p > 0.05 |
| day 80 | 37 | (2 - 159) | | 30 | (5 - 52) | 23 | | (6 - 75) | 15 | | (6 - 93) | p > 0.05 |
| day 100 | 36 | (9 - 105) | | 19 | (6 - 52) | 30 | | (7 - 70) | 18 | | (4 - 80) | p > 0.05 |
| day 140 | 51 | (7 - 132) | | 41 | (12 - 67) | 52 | | (21 - 97) | 19 | | (10 - 115) | p > 0.05 |
| day 180 | 82 | (21 - 170) | | 86 | (31 - 144) | 104 | | (37 - 216) | 108 | | (31 - 150) | p > 0.05 |
| day 240 | 176 | (82 - 283) | | 106 | (39 - 246) | 143 | | (61 - 266) | 147 | | (70 - 280) | p > 0.05 |
| day 300 | 270 | (138 - 358) | | 136 | (44 - 202) | 225 | | (107 - 376) | 130 | | (46 - 280) | p < 0.01 |
| day 360 | 316 | (236 - 413) | | 125 | (66 - 285) | 239 | | (127 - 403) | 213 | | (146 - 315) | p < 0.01 |

Median count and interquartile range of lymphocyte subsets after alloHCT.

### Supplemental Methods
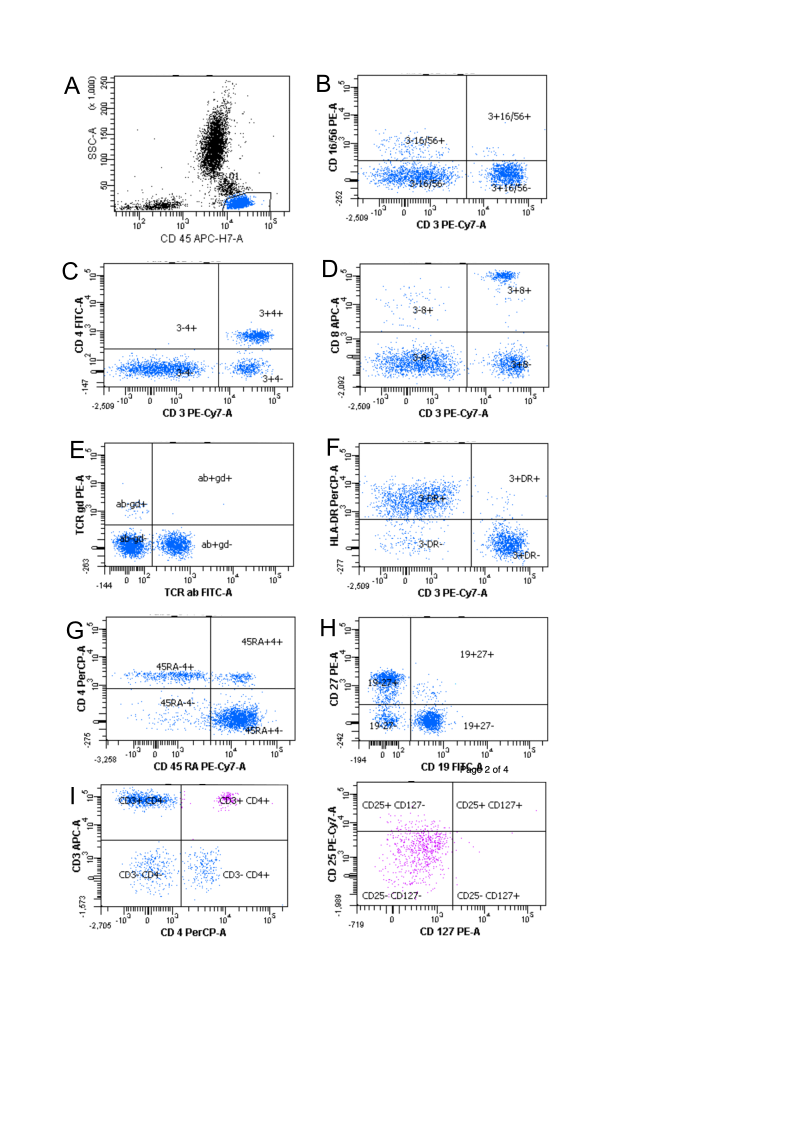


Lymphocyte subsets were determined via flow cytometry after full blood staining on an FACS CANTO II™ and analyzed with FACS Diva™ (Becton Dickinson™, New Jersey, USA). (A) Lymphocytes were identified via side scatter and CD45+. The following subgates were defined. (B) NK cells: CD3-/CD16+/CD56+, (C) CD4+ lymphocytes: CD3+CD4+, (D) CD8+ lymphocytes: CD3+CD8+, (E)  γδ T cells: CD3+ TCRγδ +/ TCRαβ -,  (F) CD3+ HLA-DR+, (G) naive T cells: CD3+ CD4+ CD45RA+, (H) B lymphocytes: CD19+, (I) Tregs: CD3+CD4+CD25+CD127-.
